# A cohort analysis of men with a family history of BRCA1/2 and Lynch mutations for prostate cancer

**DOI:** 10.1186/s12885-016-2573-x

**Published:** 2016-07-25

**Authors:** Lynne Kerr, Matthew J. Rewhorn, Mark Longmuir, Sioban Fraser, Shaun Walsh, Nicola Andrew, Hing Y. Leung

**Affiliations:** 1 Department of Urology, NHS Greater Glasgow and Clyde, Glasgow, G51 4TF UK; 2 West of Scotland Genetic Service, Queen Elizabeth University Hospital, NHS Great Glasgow and Clyde, Glasgow, G51 4TF UK; 3 Department of Pathology, Queen Elizabeth University Hospital, NHS Great Glasgow and Clyde, Glasgow, G51 4TF UK; 4 Department of Pathology, Ninewells Hospital, Dundee, DD1 9SY UK; 5 Human Genetics Unit, Ninewells Hospital, Dundee, DD1 9SY UK; 6 Institute of Cancer Sciences, University of Glasgow, Glasgow, G61 1BD UK; 7 CRUK Beatson Institute, Glasgow, G61 1BD UK

**Keywords:** BRCA1/2, Lynch Syndrome, Mismatch repair, MSH6 Mutation, Prostate cancer

## Abstract

**Background:**

Prostate cancer (PC) is a major health concern for men worldwide, with an estimated lifetime risk of ~14 %. A recent comprehensive analysis of mutational processes revealed ageing and mismatch repair as the only altered processes in PC. We wish to test if a cohort of men with inherited risk of mismatch repair defect through *BRCA1/2* or Lynch Syndrome mutations represents a target population for prostate cancer testing.

**Methods:**

Fifty-eight men (aged 40–69 years) from families with a history of BRCA1/2 or HNPCC/Lynch mutations were invited to take part. Men with PSA >3.0 ng/ml were recommended to have transrectal ultrasound-guided prostatic biopsies.

**Results:**

Overall 1 of 7 (14 %) and 1 of 20 (5 %) men with *BRCA1/2* mutations were positive for a diagnosis of prostate cancer. In men with Lynch syndrome, 1 of 4 (25 %) was diagnosed to have prostate cancer. The index case with Lynch syndrome harbours a heterozygous mutation in the mismatch repair *MSH6* gene. Near to complete loss of MSH6 immunoreactivity in the prostate tumour supports silencing of the remaining *MSH6* allele during prostate carcinogenesis.

**Conclusion:**

The finding of near-to-complete loss of MSH6 expression in affected men with a family history of Lynch Syndrome supports its mechanistic involvement during prostate carcinogenesis. This work therefore contributes to the argument that, in certain male populations, Lynch Syndrome mutations are biologically implicated in men with prostate cancer.

**Electronic supplementary material:**

The online version of this article (doi:10.1186/s12885-016-2573-x) contains supplementary material, which is available to authorized users.

## Background

Prostate cancer (PC) is the commonest solid malignancy diagnosed in men in the Western world, and the second leading cause of male cancer death. The estimated lifetime risk of prostate cancer is reported to be at ~14–18 %, with a median age of 66 years at diagnosis [1]. Genetic factors contribute to at least 40 % of all PC in patients under 50 [2].

BRCA1 and BRCA2 (breast cancer 1/2, early onset) as key molecules for the homologous recombination pathway to repair double-strand DNA breaks are intrinsically involved in the maintenance of the genome stability. Inherited mutations in the *BRCA1* and *BRCA2* genes confer increased lifetime risk of developing breast and/or ovarian cancer. Hereditary Non-polyposis Colorectal Cancer (HNPCC) or Lynch syndrome, an autosomal dominant hereditary cancer trait, carries a genetic predisposition to colorectal cancer with an 80 % lifetime risk of colorectal cancer, accounting for 1–5 % of all colorectal cancers as well as other cancers including endometrial, gastric, ovarian, urinary tract, pancreatic, brain and sebaceous tumours [3]. Collectively, men with germline mutations in *BRCA1*, *BRCA2* or *HNPCC* (Lynch syndrome) are reported to be at increased risk of developing PC [4, 5].

Most reported studies in literature have described findings in isolation on men with individual risk factors. Here, we present our cohort of men with a family history for one of the two inherited syndromes, focusing on individuals with elevated Prostate Specific Antigen (PSA) levels that warrant diagnostic prostatic biopsies.

## Methods

### Patient cohort

From 2010, with appropriate ethics and Health Board approvals, the West of Scotland Regional Genetic Service, in collaboration with local Urology Service, invited men aged 40–69 years of age from families with a history of *BRCA1/2* mutations to take part in this study. From 2013, men with a family history of Lynch mutations (*MLH1*, *MSH2*, *MSH6*, [PMS2 not included]) were also included. The reference for ethics approval for the study (as part of the IMPACT study) is 05/MRE07/25. Informed consent to participate in this study was obtained from all participants. All data presented in the report are presented in an anonymous manner in keeping with ethics approval. Men were excluded if they were known to have PC or if they had a prior cancer diagnosis with a prognosis of <5 years. Participants underwent PSA testing at enrolment, and if their PSA value exceeded 3.0 ng/ml, 10 to 12-core transrectal ultrasound– (TRUS-) guided prostate biopsies were recommended. Participants with PSA ≤3.0 ng/ml underwent annual PSA screening for ≥5 year. Participants with PSA >3.0 ng/ml and a negative biopsy will undergo annual PSA testing, repeating the biopsy if PSA increases by >50 %.

### Immunohistochemistry (IHC)

Tissue sections were processed sequentially: (1) Deparaffinisation at 72 °C, with Cell Conditioning using CC1 at 100 °C for 72 min; (2) Incubation with primary antibody for 1 h; and (3) Counterstain with Haematoxylin for 8 min and Bluing Reagent for 4 min. Standard protocol for IHC was followed using the Ventana Benchmark Ultra Machine. Individual primary antibodies were applied at the following concentrations: MLH1 (Ventana) - 1.4 μg/ml; MSH2 (Cell Marque) - 4.67 μg/ml; PMS2 (Cell Marque) - 2.19 μg/ml; MSH6 (Cell Marque) - 0.101 μg/ml. Visualisation of immuno-reactivity was performed using the Optiview Dab IHC detection Kit.

### Microsatellite instability (MSI) analysis

A multiplex PCR protocol was applied for MSI analysis. Samples for PCR were set up using the 2× Qiagen Multiplex PCR Kit to amplify five mononucleotide markers which were fluorescently labeled: BAT25(c-kit), BAT26(MSH2), NR21(SLC7A8), NR24(ZNF-2) and MONO27(MAP4K3) [6]. One microlitre of the template DNA extracted from formalin fixed tissue using the EZ1 Robot (Qiagen) was added to 25 ul PCR reaction. The following PCR programme was used to amplify selected markers: (Heated lid at 110 °C) 95 °C for 10 min, then 34 cycles of 94 °C for 1 min, 58 °C for 1 min and 72 °C 1 min, followed by 72 °C for 10 min. Following amplification, 1 ul of the PCR products were added to 0.5 ul of GeneScan™ 500 ROX™ Size Standard (an internal lane size standard for the Applied Biosystems fluorescence-based DNA electrophoresis systems) and 8.5 ul of formamide. Following denaturation (95 °C for 2 min and snap cooled on ice), the products were run on the 3130 genetic analyser (Applied Biosystems) and data processed using Genemarker software. Data supporting the conclusion of this article are included within the article.

## Results

A total of 58 men (mean age of 55.7 years, range: 42–70 years old) were recruited to the study. Eight men were initially recruited but dropped out of the study, leaving 51 informative cases for analysis: 47 men linked to a family history of *BRCA1/2* mutation and 4 men with family history of Lynch Syndrome. The mean age and serum PSA levels were comparable between men from *BRCA1/2* and Lynch Syndrome mutations (Table 1 and Additional file 1). In accordance to study protocol, prostatic biopsies were offered to individuals with PSA >3 ng/ml. Four individuals had elevated PSA levels at the time of recruitment, with a further 3 men showing a subsequent rise in PSA to exceed 3 ng/ml (Tables 2 and 3). Among the men with *BRCA1/2* mutation history, 2 of the 47 (~4.5 %) men have histological confirmation of PC with Gleason score 6 disease. Interestingly, both men were positive for BRCA mutations: 1 for *BRCA1* and 1 for *BRCA2*. None of the 19 *BRCA1/2* mutation negative cases were diagnosed to have PC (Tables 4 and 5). Overall, 1 of 7 (14 %) and 1 of 20 (5 %) men with BRCA1 and BRCA2 mutations were positive for a diagnosis of PC. Among the four men with Lynch Syndrome mutations, 1 of 4 (25 %) was diagnosed to have PC. The BRCA1 affected man has inherited a heterozygous two base pair deletion within the *BRCA1* gene (c.2681_2682delAA, Genbank accession number: NM_007294.3) which is predicted to result in premature termination of the BRCA1 protein, p.(Lys894Thrfs*8). The heterozygous *BRCA2* point mutation (c.9294C>G, Genbank accession number: NM_000059.3) identified in the patient with PC results in premature termination of the BRCA2 protein p.(Tyr3098*). These two PC sufferers with a genetic linkage of *BRCA1/2* mutations were both found to have low volume Gleason score 6 diseases, and managed by active surveillance for over 5 years without clinical evidence of disease progression.

**Table 1:** Summary of age and PSA levels of recruited individuals (*n* = 50)

Categories	Mean age; range in bracket	Mean PSA levels; range in bracket
BRCA1 +ve (*n* = 7)	55.6 (42–64)	1.37 (<0.1–4.1)
BRCA1 –ve (*n* = 7)	59.3 (43–70)	1.84 (0.6–4.1)
BRCA2 +ve (*n* = 20)	55.7 (42–71)	1.4 (0.3–4.8)
BRCA2 –ve (*n* = 12)	58.5 (44–72)	1.89 (0.2–3.9)
Lynch +ve (*n* = 4)^a^	54.5 (54–56)	3.9 (0.8–11.1)

^a^All individuals with family history of Lynch syndrome were positive for the mutation

**Table 2:** Distribution of PSA levels in the recruited individuals

PSA range (ng/ml)	<1	1–1.9	2–2.9	>3
Initial PSA	24	18	5	4
Final PSA	20	18	6	7

Initial PSA = PSA when patients were recruited; Final PSA = PSA level during the duration of this study

**Table 3:** Outcome of 7 individuals with elevated PSA levels undergoing prostatic biopsies

Histology	Benign	High grade PIN	Prostate cancer^a^
Number	3	1	3

^a^Of three patients positive for prostate cancer, all have Gleason score 6 tumour in diagnostic biopsies

**Table 4:** Summary of clinic-pathologic parameters of men diagnosed with prostate cancer in the study cohort

Clinico-pathological parameter	Patients diagnosed with prostate cancer (*n* = 3)	BRCA1 +ve case	BRCA2 +ve case	Lynch +ve case
Age	Mean 60 years old (range: 55–66)	66	60	55
PSA (ng/ml)		4.8	4.1	11.1
Gleason score	6 (3 + 3)	3 + 3	3 + 3	3 + 3
TNM stage	T_1c_-T_3_N_0_M_0/X_	T_1c_N_0_M_x_	T_1c_N_0_M_x_	T_3_N_0_M_0_
Treatment/management		Active surveillance	Active surveillance	Radical prostatectomy

**Table 5:** Summary information on recruited patients developing elevated PSA levels

	BRCA1 −ve	BRCA1 +ve	BRCA2 −ve	BRCA2 +ve	Lynch −ve	Lynch +ve
Total number	7	7	12	20	0	4
Number with elevated PSA	1	1	3	1	0	1
Number undergoing biopsies	1	1	3	1	0	1
+ve prostate cancer	0	1	0	1	0	1

**Fig. 1:**
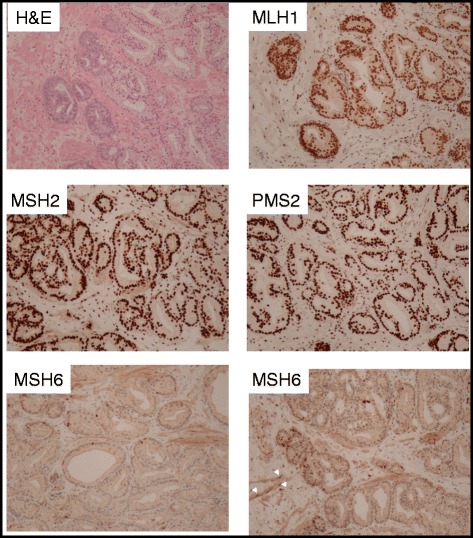
Immunohistochemistry of the prostatectomy specimen from patient with Lynch syndrome. MLH1, MSH2 and PMS2 immunoreactivity were clearly detected in the malignant epithelium. The tumour was mostly negative for MSH6 staining except for a few scattered cells. White arrows signify MSH6 expression in adjacent smooth muscle cells

The individual with Lynch syndrome, diagnosed with PC, has a familial heterozygous mutation within the *MSH6* gene (c.3939_3957dup19, Genbank accession number: NM_0001791) predicted to result in a truncated MSH6 protein product due to premature termination of the open reading frame at position 1324, p.(Ala1320SerfsX5). As expected, this patient has a family history of colorectal cancer (CRC) with three members affected by CRC. Digital rectal examination revealed a small, firm prostate with prominent left lobe. TRUS biopsy showed Gleason score 6 prostate adenocarcinoma involving 4 out of 6 biopsies from the left side of the gland. He underwent radical prostatectomy and recovered uneventfully with good urinary continence, negative (cancer free) surgical margins and undetectable serum PSA levels. His disease was pathologically upgraded to Gleason score 7 disease. Based on immunohistochemistry analysis, the key mismatch match repair (MMR) proteins, namely MLH1, PMS2, and MSH2, were expressed at normal levels, while there was a general absence of expression for the *MSH6* gene, with very occasional negligible/weak focal immunoreactivity noted within the tumour (Fig. 1). These expression data on MSH6 support a causative relationship between inherited *MSH6* gene mutation and prostate carcinogenesis, although using probes designed for colorectal cancer, microsatellite instability was not detected in the prostate tumour tissue.

Besides the above patient with PC, among the four men with Lynch Syndrome mutation, we observed a history of urological malignancy in a 40 year-old man (with a 7 base pair deletion within the *MSH6* gene) diagnosed with testicular tumours prior to recruitment to this study, right sided tumour followed by a left sided tumour 4 years later. He underwent right inguinal orchidectomy and adjuvant chemotherapy with bleomycin, etoposide and cisplatin for his right testicular tumour (non-seminomatous germ cell tumour). The left testicular tumour was found to be a mixed germ cell tumour with seminoma, yolk sac tumour and teratoma following inguinal orchidectomy.

## Discussion

The IMPACT study is a large-scale study looking at the relative risk of men with a history of hereditary BRCA mutations in developing prostate cancer at a population level. This report case cohort contributes to the overall IMPACT study cases. However, none of the prostate cancer identified within the IMPACT study would have undergone investigations to test if the mutated gene was in fact functionally altered in relation to the development of prostate cancer. In our report, we carried out molecular investigation to confirm the presence of mutations in the affected individuals as well as characterised the expression levels of the implicated gene (MSH6). We found that there was evidence of loss of expression for this mismatch repair gene for Lynch Syndrome at the protein level, which provides indirect mechanistic evidence to support the presence of molecular event that results in the lost of gene expression at protein level, in addition to the presence of an inherited mutation in the mismatch repair enzyme gene. A single mutation in a heterozygous manner would not typically affect the gene expression as drastic as what we have observed. In addition, within the recent published IMPACT series [7], patients with a history of Lynch Syndrome were not included for analysis. Hence, it remains highly debated whether men affected by Lynch Syndrome are at risk of developing prostate cancer. Our report, albeit small, contributes to the literature in this field. Important publications arguing for an increased risk for prostate cancer with Lynch Syndrome are appropriately cited in our report.

Besides changes in genetic profile associated with ageing, a recent comprehensive analysis of mutational processes in human cancer has revealed mismatch repair mutations to be the only genetic signature implicated in PC [8]. Among the mismatch match repair (MMR) genes, defects within the *MSH2* and *MSH6* genes have been previously reported in patients with PC [9]. In a prospective unbiased analysis of a cohort of 446 unaffected carriers of an MMR gene mutation (MLH1, MSH2, MSH6, and PMS2) and 1029 of their unaffected relatives, as part of a Colon Cancer Family Registry, MMR gene mutation was found to confer increased cancer risks in a number of tumour types recognised to be associated with Lynch Syndrome (colorectal, endometrial, ovarian, renal, gastric, and urinary bladder), breast cancer and pancreatic cancer [10]. Importantly, the non-carrier relatives of family-specific MMR gene mutations did not have increased risks of colorectal or other cancers, including PC. Bratt et al. identified a two fold increased risk of PC associated with *MSH2* mutation [11]. MMR mutation carriers were found to be nearly 6 times more likely to develop PC [12]: PC occurred at an earlier age than expected (60.4 years compared with 66.6 years) and tumours were significantly more aggressive (Gleason 8–10).

BRCA1 and BRCA2 are implicated in homologous-recombination based DNA double-strand break repair. BRCA1 is also implicated in mismatch repair, interacting with the DNA mismatch repair protein MSH2 [13, 14]. In addition to the genomic structural instability conferred by defective double-strand break repair, a base substitution mutational signature in keeping with microsatellite instability, was also associated with BRCA1/2 deficiency [8].

The cumulative lifetime risk of PC among carriers with mutations in MMR-related genes has been suggested to be as high as 30 %, compared with ~18 % in the general population [9]. Our patient cohort is included in the recently published data on the initial screening round of the IMPACT [15] (Identification of Men with a genetic predisposition to ProstAte Cancer: Targeted screening in *BRCA1/2* mutation carriers and controls; www.impact-study.co.uk) Study, supporting the notion that men with *BRCA1/2* gene mutations are at increased risk of developing PC. Overall, from the 2481 recruited men within the IMPACT study [15], 199 men had PSA >3.0 ng/ml, and 162 men underwent prostatic biopsies. Among biopsied patients, 59 (or 36 % of) men were found to have PC. Similarly, in our cohort, 3 of 7 (or 43 %) have positive biopsies for PC. While 66 % of the PC patients within the overall IMPACT study have intermediate- or high-risk disease, all three patients with PC have relatively low-risk (Gleason 6) disease at diagnosis, although the single patient undergoing prostatectomy was found to have his disease upgraded to Gleason 7 tumour. This data contrast a recent report suggesting that BRCA mutations were associated with less favouable outcome following radical treatment [7].

Our study supports the notion that men with a family history of *BRCA1/2* or Lynch Syndrome mutations may benefit from testing for PC, highlighting the role of MMR in prostate carcinogenesis. Specifically, our patient with documented *MSH6* mutation and associated loss of its expression involves a less commonly implicated member of the MMR genes [16].

## Conclusion

The finding of near-to-complete loss of MSH6 expression in affected men with a family history of Lynch Syndrome supports its mechanistic involvement during prostate carcinogenesis. This work therefore contributes to the argument that, in certain male populations, Lynch Syndrome mutations are biologically implicated in men with prostate cancer.

## Additional file

Additional file 1: Tables showing patient details according to the genetic background. (DOCX 184 kb)

## Abbreviations

PC: Prostate cancer
BRCA1 and BRCA2: breast cancer 1/2, early onset
HNPCC: Hereditary Non-polyposis Colorectal Cancer
PSA: Prostate Specific Antigen

## References

[1] Howlader N, Noone AM, Krapcho M, Garshell J, Miller D, Altekruse SF, et al. SEER Cancer Statistics Review, 1975–2012, National Cancer Institute. Bethesda, MD, http://seer.cancer.gov/csr/1975_2012/, based on November 2014 SEER data submission, posted to the SEER web site, April 2015

[2] Chen Y, Wang J, Fraig MM, Henderson K, Bissada NK, Watson DK (2003). Alterations in PMS2, MSH2 and MLH1 expression in human prostate cancer. Int J Oncol.

[3] Roupret M, Yates DR, Comperat E, Cussenot O (2008). Upper urinary tract urothelial cell carcinomas and other urological malignancies involved in the hereditary nonpolyposis colorectal cancer (Lynch Syndrome) tumour spectrum. Eur Urol.

[4] Ryan S, Jenkins MA, Win AK (2014). Risk of prostate cancer in Lynch syndrome: a systematic review and meta-analysis. Cancer Epidemiol Biomarkers Prev.

[5] Castro E, Goh CL, Eeles RA (2013). Prostate cancer screening in BRCA and Lynch syndrome mutation carriers. Am Soc Clin Oncol Educ Book.

[6] Bacher JW, Flanagan LA, Smalley RL, Nassif NA, Burgart LJ, Halberg RB (2004). Development of a fluorescent multiplex assay for detection of MSI-High tumors. Dis Markers.

[7] Bancroft EK, Page EC, Castro E, Lilja H, Vickers A, Sjoberg D (2014). Targeted Prostate Cancer Screening in BRCA1 and BRCA2 Mutation Carriers: Results from the Initial Screening Round of the IMPACT Study. Eur Urol.

[8] Alexandrov LB, Nik-Zainal S, Wedge DC, Aparicio SA, Behjati S, Biankin AV (2013). Signatures of mutational processes in human cancer. Nature.

[9] Raymond VM, Mukherjee B, Wang F, Huang SC, Stoffel EM, Kastrinos F (2013). Elevated risk of prostate cancer among men with Lynch syndrome. J Clin Oncol.

[10] Win AK, Young JP, Lindor NM, Tucker KM, Ahnen DJ, Young GP (2012). Colorectal and other cancer risks for carriers and non carriers from families with a DNA mismatch repair gene mutation: a prospective cohort study. J Clin Oncol.

[11] Bratt O (2000). Hereditary prostate cancer. BJU Int.

[12] Grindedal EM, Moller P, Eeles R, Stormorken AT, Bowitz-Lothe IM, Landrø SM (2009). Germ-line mutations in mismatch repair genes associated with prostate cancer. Cancer Epidemiol Biomarkers Prev.

[13] Wang Q, Zhang H, Guerrette S, Chen J, Mazurek A, Wilson T (2001). Adenosine nucleotide modulates the physical interaction between hMSH2 and BRCA1. Oncogene.

[14] Peng M, Xie J, Ucher A, Stavnezer J, Cantor SB (2014). Crosstalk between BRCA-Fanconi anemia and mismatch repair pathways prevents MSH2-dependent aberrant DNA damage responses. EMBO J.

[15] Castro E, Goh C, Leongamornlert D, Saunders E, Tymrakiewicz M, Dadaev T (2015). Effect of BRCA mutations on metastatic relapse and cause-specific survival after radical treatment for localised prostate cancer. Eur Urol.

[16] Rosty C, Walsh MD, Lindor NM, Thibodeau SN, Mundt E, Gallinger S (2014). High prevalence of mismatch repair deficiency in prostate cancers diagnosed in mismatch repair gene mutation carriers from the colon cancer family registry. Familial Cancer.

