# Supplementary figures and images for: A cohort analysis of men with a family history of BRCA1/2 and Lynch mutations for prostate cancer
Source: BMC Cancer. 2016 Jul 25;16:529. doi: 10.1186/s12885-016-2573-x (PMC4960816; doi:10.1186/s12885-016-2573-x)

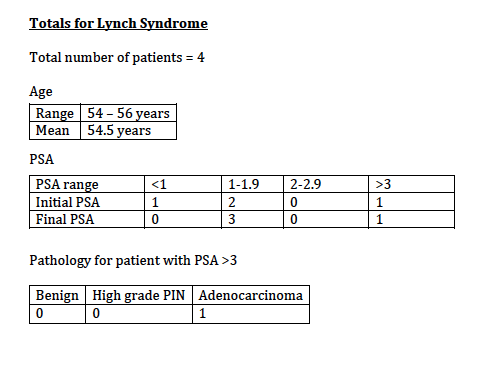


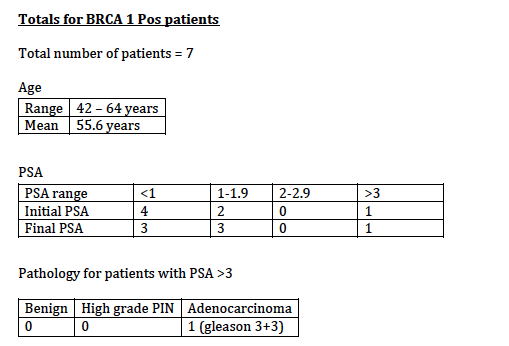


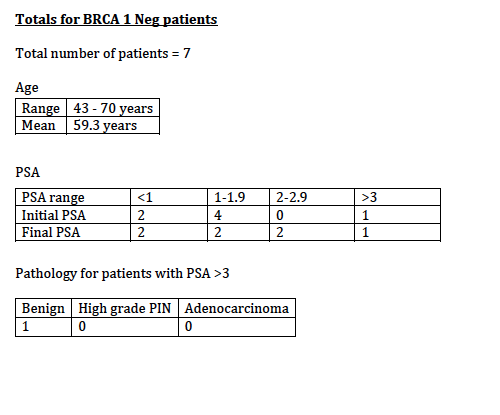


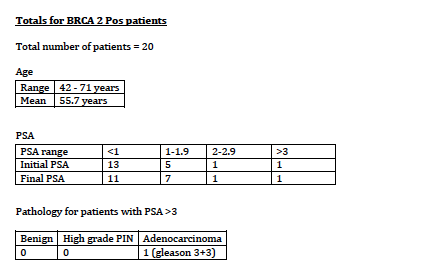


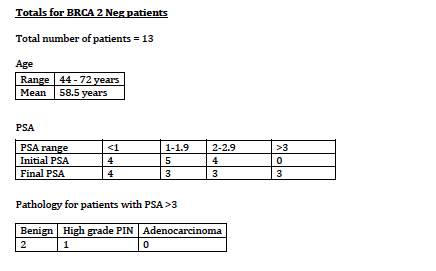

Supplement: Additional file 1: — Tables showing patient details according to the genetic background. (DOCX 184 kb) [file 12885_2016_2573_MOESM1_ESM.docx]
